# The Yellow Fever Commissions

**Published:** 1879-03-20

**Authors:** 


### *THE YELLOW FEVER COMMISSIONS.*

There were three Yellow Fever Commissions chosen to investigate the late epidemic of yellow fever. The expenses of the two commissions were borne by a liberal-minded lady, Mrs. Elizabeth Thompson, of New York. Their reports have been discussed generally and with many unfavorable criticisms in the journals and newspapers. The united report of the Congressional Commission and the New Orleans Commission we refer to under head of Book Notices. We have received also the Special Report of the Homœopathic Commission. This Commission agrees as to the fact that the disease is indigenous to the West Indies and perhaps to the West coast of Africa, but claims that the yellow fever germs have been thoroughly naturalized in many localities in the Southern portion of the United States.

